# “Should I tell him I have something in my vagina?” Female sex workers’ perceptions and experiences of using a menstrual cup, and client reactions: A qualitative study in Western Kenya

**DOI:** 10.3389/frph.2025.1740096

**Published:** 2026-02-02

**Authors:** Linda Mason, Edyth Osire, Enid Awiti, Cynthia Akinyi, Clarah Akello, Garazi Zulaika, Fredrick O. Otieno, Pennelope A. Phillips-Howard, Supriya D. Mehta

**Affiliations:** 1 Department of Clinical Sciences, Liverpool School of Tropical Medicine, Liverpool, United Kingdom; 2 Nyanza Reproductive Health Society, Kisumu, Kenya; 3 Centre for Global Health Research, Kenya Medical Research Institute (KEMRI), Kisumu, Kenya; 4 Division of Infectious Disease Medicine, Rush University College of Medicine, Chicago, IL, United States

**Keywords:** clients, female sex workers, Kenya, menstrual cup, menstrual disc, qualitative

## Abstract

**Introduction:**

The menstrual cup is worn intravaginally, holding blood up to 12 h before emptying and reinserting. It offers protection from sexually transmitted infections and bacterial vaginosis, whilst preserving a *Lactobacillus crispatus*–dominant vaginal microbiome. The menstrual disc, a type of menstrual cup, is positioned near the cervix and can remain in place during sex, enabling female sex workers (FSWs) to avoid unsafe practices to conceal menstruation during work. In this study, we aim to examine the perceptions and experiences of a convenience sample of FSWs 6 months after they received a menstrual disc, along with client views.

**Methods:**

In a qualitative design, our Kenyan study recruited 38 FSWs in 4 focus group discussions (FGDs) and 86 clients in 9 FGDs. Using a semi-structured guide, a Kenyan moderator and note-taker facilitated audio-recorded discussions. Following translation and transcription, the discussions were analysed using deductive thematic analysis.

**Results:**

Six themes emerged: Anticipation and reaction to seeing the menstrual cup, Apprehension and determination to use, Benefits, Challenges, Secrecy, and Use during sex. Some FSWs were able to insert the cup on initial attempt, typically others encountered discomfort, pain, or misalignment during insertion or difficulty in removing. By the end of the third month, the majority were using the cup without experiencing any leakage, pain, or concerns. Benefits noted included ease, convenience, reduced leakage, and comfort. Financial advantage over pads and ability to work regularly were also noted. Nearly all FSWs used the cup during sex, despite prior anxiety that a client would detect it and react negatively. In three instances, the FSWs reported that a client “may” have felt the disc, whilst noting just two clients “may” have felt something. None reacted badly. In summing up their experience, the FSWs spoke very positively about the disc, with the intention to keep wearing it in the future. Many clients were supportive of the disc but did not want to know whether an FSW was wearing it.

**Conclusions:**

The FSWs quickly adapted to using the disc, finding it a comfortable, reliable menstrual product with financial advantages. With clients remaining mostly unaware of its use, the FSWs enthusiastically embraced the disc with the intention to keep using it. These positive outcomes have implications for future scale-up and roll-out to other vulnerable female populations.

## Introduction

The importance of good menstrual health and hygiene (MHH) is acknowledged as a fundamental right of girls, women, and other menstruators, which is essential for them to reach their full potential (1). Good MHH is dependent on a range of factors, including knowledge and a supportive environment to manage menstruation, access to water sanitation and hygiene (WASH) facilities, and affordable and quality menstrual hygiene products.

Currently, there are an array of commercially produced menstrual hygiene products such as sanitary pads, liners, tampons, period pants, and menstrual cups. In this study, we focus on a specific type of menstrual cup, called the “menstrual disc.” The menstrual cup is a sustainable and environmentally friendly option compared with disposable pads and tampons. Menstrual cups are bell- or disc-shaped receptacles made of silicone, natural rubber, or thermoplastic elastomers (TPEs) that are worn intravaginally and hold menstrual blood. They can remain *in situ* for up to 12 h before being emptied and reinserted. Relative to other commercially available products, they entail higher initial financial expense, but they can last up to 10 years, becoming a cost-saving option within 6–12 months (2). Cases of toxic shock syndrome (TSS) are extremely rare when menstrual cups are used, with a much lower incidence compared with tampons (2). However, there is a minor risk of dislodgement when the cup is used in conjunction with an intrauterine device (IUD), and therefore, medical advice should be sought first. Apart from environmental and economic advantages, recent research suggests that menstrual cups may offer protective effects against sexually transmitted infections (STIs) and bacterial vaginosis (BV) (3–5), with menstrual cup use preserving a *Lactobacillus crispatus*–dominant vaginal microbiome (3).

A meta-analysis of 13 studies showed high acceptability for menstrual cups, with three-fourths of participants reporting that they wished to continue use at study end (2). Comparisons with other menstrual products suggest that many users prefer cups to pads or tampons. A Canadian randomised trial of menstrual cups vs. pad/tampon reported that cups were better in terms of convenience and non-leakage, and that 91% would continue using the menstrual cup and recommend to others (6). A qualitative study in Kenyan schoolgirls noted that girls who had tried both cups and commercial pads preferred cups for reasons of comfort, better movement, and no fear of leakage (7). Similar findings were reported in a recent Indian study amongst adult menstrual cup users aged 25–37 (8).

However, no studies to date have specifically evaluated the use of the menstrual disc that sits high against the cervix, with a lip rather than a tail or stem to facilitate removal. Unlike the vaginal cup, because of its high positioning and lack of protuberance, the disc can be left *in situ* during sex. Coupled with its biological protective effects, it may be particularly advantageous for women who have sex for livelihood. The few studies to date indicate that many female sex workers (FSWs) lose their income by not being able to work during menses, or resort to hiding their menses from clients (9, 10). Women report attempting to hide menstrual blood by inserting cotton wool, tissue, or other material into the vaginal canal, and this practice is associated with discharge, chronic vaginal infection, and malodour (9). Lees et al. (11) report that women in Uganda and Tanzania resort to insertion of herbs, soda (e.g., Coca-Cola), lemon juice, and beer amongst other items to cleanse and remove “dirt,” including that of menstrual blood, and to stem flow prior to sex. Vaginal douching has been associated with increased BV risk (12). When unsuccessful in hiding their bleeding, FSWs have reported about male clients refusing to pay or becoming violent.^1^ These narratives have been confirmed in a study of male clients of FSWs in Western Kenya, with the majority of whom also reporting that menstruating woman are unclean or dirty (10).

There is an important need to undertake an evaluation of the menstrual disc, including its acceptability as a menstrual solution during sex. In this study, we present findings from a qualitative study undertaken to explore the views and experiences of FSWs about the menstrual disc, including during use with clients. We also contextualise with male client experiences and their knowledge of and views on FSWs wearing a menstrual disc during their sexual encounters.

## Materials and methods

### Setting

The study was conducted in Kisumu, Western Kenya, situated on the shores of Lake Victoria approximately 320 km from Nairobi. The population is predominantly of the Luo tribe. Roughly half of the population lives in informal settlements (13). This area has a high HIV rate, with over 15% of the general adult population diagnosed with HIV/AIDS (14). Although sex for economic livelihood is criminalised in Kenya, a 2017–2018 nationwide assessment suggests that there are over 5,000 women engaged in sex for livelihood in and around Kisumu City (15).

### Study design and sample

This qualitative study is nested in a single-arm trial amongst FSWs to evaluate the safety and effectiveness of a reusable menstrual disc that can be inserted during sex (16). In this study, we examine the perceptions and experiences of a convenience sample of FSWs at approximately 6 months after they receive a menstrual disc. We also include data from focus group discussions (FGDs) with male clients of these FSWs who were interviewed primarily for an investigation into their changing relationships with these women at times of economic stress, but from whom we took the opportunity to ask about their knowledge, experience with, and views on having sex with an FSW who was wearing a menstrual disc to provide a full account of user and client perspectives of the menstrual disc. Content from the pertinent sections of the transcripts was analysed to add full perspectives for this paper.

### Recruitment and procedure

We chose to collect data by using FGDs as FSWs are part of a close-knit community and would therefore likely contribute to a lively discussion. Similarly, a previous experience of conducting FGDs with male clients had provided a wealth of data..

Aiming for approximately 8 participants per FGD, with each 50 FSWs provided with their disc, a simple random sample of 12 participants was drawn, assuming that some women may not want to participate in the FGDs or may not be available. Each participant listed was invited by phone to participate in an FGD by the study coordinator. Names were added to the list if fewer women were recruited. We aimed to conduct six FGDs at the outset; however, after each FGD, a debriefing discussion was held with the moderator, note-taker, PI, and lead author, which noted key findings and suggested possible refinements to the semi-structured guide or further prompts. After three FGDs, we noted that no new findings emerged; but conducted a fourth FGD to confirm data saturation had been reached.

Male participants who were clients of FSWs were recruited using convenience sampling, deemed most feasible given the nature of the target population. Peer educators and FSWs enrolled in the trial helped identify potential male clients from designated hotspots (e.g., bars, brothels, guesthouses, and streets). These clients were approached by peer educators who shared their contact details with the study coordinator if they were amenable to participating in the FGDs. We aimed for eight participants per group, but we oversampled due to potential under-attendance. A total of nine FGDs were conducted with clients of the FSWs. Whilst we found that data saturation was reached very quickly pertaining to information required for the purpose of this paper (four FGDs achieved this), our primary data objective required additional data collection. We included relevant data from all nine FGDs within the current analysis.

All FGDs took place in a private room. Written informed consent was obtained from each participant in their preferred language (English, Dholuo, or Kiswahili), including explicit permission for audio recording. Prior to beginning the discussion, participants were reminded of the study purpose and what their involvement would entail. They talked through and agreed on a set of ground rules, including the need for confidentiality and to respect each other's opinions. Anonymity of the recordings was preserved by using a participant number that each participant was asked to state as they began speaking. The female project coordinator/moderator and female note-taker who led all FSW FGDs were fluent in English, Dholuo, and Kiswahili, and participants were given the option of speaking in whichever language they were most comfortable with. Similarly, the moderator and note-taker for the male client FGDs were fluent in all three languages, and the participants were similarly free to speak in their preferred language. The moderator led the FGDs using a semi-structured guide. The key questions and prompts were developed to assess the thoughts and experiences of FSWs on menstrual cup use and cup use during sex (Supplementary File S1), and for clients, the key questions and prompts were designed to elicit knowledge of having had sex whilst a disc was being used, what their experience of it was, and their thoughts on it being used and informed about it (Supplementary File S2).

FGDs were digitally recorded then transcribed verbatim using a local transcription service. Each transcript was checked for accuracy whilst listening to the recordings. Any Dholuo and Kiswahili transcripts or sections were translated into English, with a sample back-translated for a consistency check.

### Data analysis

The analysis began by reading and re-reading the transcripts for familiarity. The transcripts were imported into NVivo v14 for organisational purposes. The data were explored using thematic analysis (17), chosen because of its flexibility in supporting interpretation of data, allowing themes to be linked with the research questions with transparency and reproducibility, and suitability for projects involving teamwork. A deductive approach was used as we intended the analysis to inform specific study objectives. We analysed the FSW data first and then we analysed the client data separately using the same techniques and triangulated findings in the discussion where relevant. The initial coding frame included codes identified *a priori* comprising key question responses and from themes identified during the debrief (discussions that took place after each FGD) Further codes were added for new detail and information arising from the participant narratives. All transcripts were coded against this frame with new codes added in the process as needed. After all codes were identified, they were grouped into major themes and sub-themes. Using these themes and sub-themes, a narrative was written with a check to ensure where shared features, consistent patterns, and deviant examples were noted. The narrative was supported using illustrative quotes. The transcripts were then re-read for interrogation purposes to ensure that the narrative represented what the authors deemed was a representative portrayal of the data, and that responses to our research questions were reliable. Checks were made to ensure that views were not under-represented or over-exaggerated and amended where necessary.

### Reflexivity

The first and lead authors/analysts acknowledged their positionality as senior academics at Western Universities and had non-Kenyan backgrounds, which had the potential to bias their interpretation of the study findings. We reduced the possibility of such bias through the following ways. All data collection was undertaken by a Kenyan moderator and note-taker. After each FGD, a debrief was held with the moderator, note-taker, first author, and lead author to discuss the findings and their interpretation. The first author then held several meetings with another member of the team FOO, a male Kenyan, to facilitate her understanding and interpretation of all client transcripts. Member checking was conducted at FSW peer review meetings, where the findings were discussed and verified and any queries resolved. Data source triangulation was conducted during the analysis, with notes made during the FGDs. The moderator and note-taker reviewed and concurred with the final draft, making minor suggestions for amendments.

## Results

A total of 38 women with an age range of 22–37 took part in four FGDs. All were Kenyan, and most belonged to the Luo ethnic group. Approximately half had completed primary education, and a further 45% had completed secondary or tertiary education. The range of years as a sex worker varied with a minimum of 2 years and a maximum of 20 (Table 1).

**Table 1:** Participant characteristics—female sex workers.

Characteristic	FGD1	FGD2	FGD3	FDG4	Total No. (%)
No. women	9	9	11	9	38 (100)
Age range	22–36	26–36	23–37	24–37	22–37
18–24	1	1	2	1	5 (13.2)
25–29	5	2	3	2	12 (31.5)
30–34	1	5	3	4	13 (34.2)
35–39	2	1	3	2	8 (21.1)
Ethnic group					
Luo	7	8	11	9	35 (92.1)
Luhya	1	1	0	0	2 (5.3)
Kikuyu	1	0	0	0	1 (2.5)
Schooling completed					
None or < primary	0	0	1	0	1 (2.6)
Primary	6	4	5	5	20 (52.6)
Secondary	2	4	4	2	12 (31.6)
Vocational/college/university	1	1	1	2	5 (13.2)
Children					
Yes	8	9	10	9	36 (94.7)
No	1	0	1	0	2 (5.3)
Years engaged in sex work, range	2–20	4–20	2–15	5–20	2–20

A total of 86 male clients with an age range of 19–49 participated in 9 FGDs. All were Kenyan, and the majority of them belonged to the Luo ethnic group. More than two-thirds of them had completed either secondary or tertiary education. Over half of them were currently married or with a partner and also did not have a regular income (Table 2).

**Table 2:** Participant characteristics—male clients.

Characteristic	FGD1	FGD2	FGD3	FDG4	FGD5	FGD6	FGD7	FGD8	FGD9	Total No. (%)
No. men	7	10	11	10	9	9	10	8	12	86 (100)
Age range	26–47	27–39	26–49	23–49	25–39	25–49	19–40	28–49	25–41	19–49
18–24	0	0	1	3	0	0	2	0	0	6 (6.9)
25–29	1	2	2	1	4	5	4	2	6	27 (31.4)
30–34	3	2	3	3	2	1	1	2	2	19 (22.1)
35–39	2	6	4	0	3	1	2	1	1	20 (23.3)
40–44	0	0	0	0	0	1	1	0	3	5 (5.8)
45–49	1	0	1	3	0	1	0	3	0	9 (10.4)
Ethnic group										
Luo	7	9	11	8	7	8	7	7	10	74 (86.0)
Luhya	0	1	0	2	2	1	2	0	1	9 (10.5)
Other	0	0	0	0	0	0	1	1	1	3 (3.5)
Schooling completed										
None or < primary	0	0	1	0	0	0	0	0	3	4 (4.7)
Primary	3	2	1	4	1	1	2	3	3	20 (23.3)
Secondary	2	4	5	3	5	7	5	1	3	35 (40.7)
Vocational/college/university	2	4	4	3	3	1	3	4	3	27 (31.4)
Marital status										
Never married	2	2	3	2	1	2	5	2	3	22 (25.6)
Currently/partner	5	6	7	6	6	7	4	3	5	49 (57.0)
Divorced/widowed	0	2	1	2	2	0	1	3	4	15 (17.4)
Regular income										
Yes	7	7	7	3	4	3	4	3	1	39 (45.3)
No	0	3	4	7	5	6	6	5	11	47 (54.7)

Seven themes were identified from the FSW analysis; Anticipation and initial reaction, Apprehension and determination, Benefits, Challenges, Use during sex, Secrecy, and Sensitisation. Findings from the client analysis were added to the following themes: Anticipation and initial reaction, Use during sex, Secrecy, and Sensitisation. Please note that all participants were provided with a disc but continued to refer to it throughout as a “menstrual cup”.

### Anticipation and initial reaction

All FSWs were first made aware of the menstrual disc when they were recruited to the study, and prior knowledge of it was rare. Most were curious, even eager to see it, primarily because of its name; others were doubtful wondering how the disc (which participants called a “cup”) could be utilised as a menstrual absorbent.

“the first time it was found weird I didn’t know then that word cup … I was wondering ee a cup … because we know what cups are. We didn’t expect that it was something nice that we can insert in our vagina” (R1 G2).

Reactions on seeing it varied from remarking on how small it was to being “afraid”—musing how it could fit into their vagina. A few participants anticipated pain, although others were immediately reassured because the cup was smaller than they had anticipated.

Despite some clients having been told about the menstrual disc, or even shown one by their female sex worker, many men seemed not to know anything about it but were curious for more information. One group (FGD7) had no clients who had heard about it or seen it. Most men had a positive response to the information and the disc itself and asked many questions of their moderator.

### Apprehension and determination

Some FSWs spoke of being able to insert and wear the menstrual disc comfortably at their initial attempt. Typically, across all FSW FGDs, women were apprehensive to insert it, encountering difficulties on insertion the very first time but either continued to try until succeeding or waited to attempt again during their next menses. A few FSWs reported reaching out for help outside of the monthly study phone calls and either spoke to one of their peers or with one of our project staff who gave instructions. A couple reported asking a peer to insert it for them. The overwhelming sense voiced by FSW was that they were determined to master and be able to wear the menstrual disc within the first few tries. Most described how after a few attempts it just seemed to “go in” without a problem, and from then on, they were comfortable and able to use with ease. Some mentioned with hindsight that they had either been inserting it incorrectly or had been too tense. A few women spoke of having difficulties in removing the disc, some of whom reached out for help. Once given advice such as positioning themselves better or relaxing, or occasionally receiving physical help from a colleague or friend, they were able to remove it. Only a couple of women stated that it wasn't until the third month that they were able to use it comfortably. This was the longest duration that any woman reported needing to become confident in disc insertion and removal. The following excerpts illustrate participants' initial experiences of using the disc.

“Day one, I felt like the heavens would fall on me, it also felt like someone was looking at me, I was disturbed the whole day, but the second day I started practicing myself and it went in, in fact I had sex with it…” (R3 G1).

“First month I never used it I was very scared, second month I tried using it and removed it, I was not comfortable when sitting like this when in my periods would thing stain my, my dress. I said to myself that I was not comfortable let me go and remove it, blood had started collecting in it, I went and bought Always [sanitary pads] from the shop. Third [month] I put it and just felt comfortable with it and I had put it at night and didn’t feel anything and I was just ok so up to now I am using it and I am enjoying using it”. (R6: G2).

“Now, at first I had a challenge, it refused to go in. I lifted my legs as you told us, I tried to insert it and it refused. Now I went and called one of us whom we were taught with. And I lifted my legs like this, that’s when she now inserted it for me, I just heard a sound of it puup! Then later I walked with it, I walked with it and I didn’t feel anything but it’s … good”. (R2 G4).

“I tried to put it on day one and it didn't go, mmm, I went back to my pads that am used to. I tried again in day two and it started leaking, mmm, I left it. Second month the same experience, in fact, it stressed me up for three months. And I now said that … mmm, today I want to try for the last time then now leave it. But the way I tried it has not bothered me again” (R3 G4).

“For me, it was getting inside with ease and filled, and I would remove it like we were told to remove it. Me, I didn’t feel anything!” (R1: G2).

### Benefits

Once insertion and removal were mastered, the FSWs agreed that the disc was easy and convenient to use, with many using the word “comfortable”. This mostly referenced physical comfort, typically like “This cup, am just comfortable with it” (R8 G3), but also sometimes indicated psychological comfort, indicating that they were free from the fear of, or having to check for, leaking, staining, or odour. Frequently, the participants related their comfort of wearing the cup to using a pad, and in every instance, they favoured the disc. The following quotes, similar to the above excerpts, are typical of narratives across all groups.

“I like the cup just because it’s comfortable, after you’ve put it on you don’t have any stress that it will leak. Before, it used to stress me up” (R3 G4).

“You cannot compare the cup to pads, for the pads when you stand, you will ask someone to check for you in case you stain your dress, but for the cup, you are comfortable and mostly when we are in our periods. We used to walk with lessos [a fabric wrap] when using pads just in case you stain your dress you have something to wrap yourself” (R8 G1).

“When I put on pads I kept on scratching myself and I was like … it was itching even in front of the people. But menstrual cup has now come, at least it’s now easier” (R9 G4).

“Okay the heat that pads bring, then these places had swellings like boils, rashes, boils but now am just good to go no more boils, no more heat rash” (R4 G2).

A few talked about improvement in their hygiene through not now having to conceal bleeding from clients by using cotton wool or tissue (locally known as ‘kasunda’) inserted into their vagina to soak up the blood:

“ I am fresh because when I placed cotton, sometimes if you do not place it well it displaces on the side of the vagina (laughter) that’s when you want to keep turning your finger inside, this one is good once placed you have nothing to worry about” (R1 G2).

“Now when you are using cotton, that cotton, your hands are dirty, with cotton you also want to push it inside there using your hands … and that’s where people usually get more infection” (R2 G3).

The cup was also positively viewed in relation to sanitary pads when the participants were asked about the financial impact. They spoke of saving money that they would have spent buying pads previously. A few highlighted that the money they saved was now being spent on their children.

“I save money that I used to buy pads with, now I buy for my daughter pads, then I used to buy for her one or two now I can buy for her that bulk. So it lasts” (R7 G2).

“I can save that money, I pay school fee for my child, I can even buy something for the child … even something that can help him” (R11 G3).

Some other women spoke of another financial benefit. Although some women had previously used *kasunda* and/or douching to hide or wash away their menstrual blood, others did not take clients while they were menstruating. Now with menstrual discs, they had a hygienic and comfortable method to keep working and thus earning money throughout their menstrual period.

“and another thing to add, the other girls at the hot spots, they are wondering we are at work Monday to Monday. (laughter) Before you could bleed for one week, one week you are broke, you don’t have money, but now I do [sex work] from 1st to 1st” (Rx G2).

### Challenges

Apart from initial issues in inserting and removing the disc, a few challenges in using it were acknowledged by FSWs when they were specifically asked about them. One report spoke of a woman losing her disc and another told of having her disc stolen. Women in three FGDs shared that they had accidentally dropped their disc on occasion, although none had in the other FGD. One woman spoke of how this occurred “many times,” and she reported, “It is an issue, the reason is, you are not accurate. You are supposed to be accurate when you are inserting it” (R1 G3). Other instances of dropping arose from being anxious when first using the disc, being in a hurry, or having had a few alcoholic drinks. A couple of women spoke of keeping the disc in for longer than the recommended period (i.e., for more than 12 h), when they had drunk too much alcohol or taken drugs. “Ahhh! Okay, I chewed, black[ed]out at two am and I woke up at nine pm in the evening” (R1 G3).

Others reported that they did not use their cup when they were going out drinking because they were worried about dropping it, or they feared forgetting they were wearing it and thus keeping it in for too long.

“normally get very drunk [laughter] but when I am going to make money I do not get drunk” [laughter] (R4 G1). “The reason I fear is because we were taught that if one is likely to get drunk, they can forget that they had the cup inside them, so were told to avoid using it in such cases, so I have never used it when drunk”

Alternatively, others concluded, “So you don't drink a lot, you control.” Her colleague, agreeing, added “If you are using it you don't drink too much” (both Rx G2).

Another stated firmly, “If am going to drink and am on my menses, I cannot have a client, I just drink my own money, that's my day to enjoy. It is very clear. So you cannot combine alcohol, combine cup, and combine with the client, you will die. [Laughter] I don't cheat you, you will die” (R7 G4). Her colleague added, “When am drunk client is no because somebody can play some games that are …” (R4 G4).

A few spoke of challenges in finding water to wipe the cup after changing, particularly if they were working or sleeping on the streets, and stressed the need for more provision of public washrooms. Yet, others reported taking water and/or wipes with them with seemingly little difficulty. A greater challenge appeared to be finding privacy to remove and empty the cup.

“I got bothered when I went to work in [venue]. I was there and I was a visitor, and you know sex work is not easy, you are in a strange place just to solicit to get money. I got a challenge because I did not get a client, I never got somewhere to sleep, I just slept on the street till the following day at 2pm. I never even got somewhere to bathe. I had the cup on till I felt some heaviness in my abdomen, I then found a lady who helped me get a place to shower and I also removed the cup. I had dizziness and abdominal discomfort. I over stayed with it” (R4 G1).

“Sometimes public toilets may be available but you do not know where they are. Like where we are, I do not know where I may go to when pressed. And if one would not be in her periods, one can just squat and pass urine, but with periods, if some sees you removing the cup, one may come after you [laughter]” (Rx G1).

Planning in advance when and where to empty was suggested by others as a way to mitigate this issue.

### Use during sex

The participants were asked whether they had sex while wearing the disc. Four women admitted that they had not tried it. One stated, “Because I'm afraid, I feel that when … that man will … whatever inside there. I always just wonder that it will get in and get lost” (R4 G4), while her two colleagues in the same group also admitted that they had not used it during sex because they were “scared.” Indeed, numerous FSWs spoke of being scared the first time they had sex whilst wearing the disc. A couple of participants said that they were worried that it would be displaced, and another said that blood would be pushed back “where it came from,” but most were concerned that the partner would feel it.

Consequently, many chose their partner carefully the first time they had sex while wearing the disc, either a boyfriend or a regular client. A couple of women instructed their partner to go slowly or gently on the pretext that they were not feeling very well. One woman had a ready response that it was a “family planning method” should the partner be able to feel it. After feeling perturbed initially, women were relieved to find that men did not feel it. While a few women asked their partner afterwards whether they had felt anything, and were told “no,” others came to the assumption that as nothing had been mentioned then the cup had not been sensed by their partner as their partner mentioned nothing about the cup or any such device.

“My first experience, that was on the second month, second day but I made sure I went with someone am used to, because I was like if I go with a new client you never know you can get a long penis. I had that in my thoughts, it can push it inside but for the first time I had sex with it inside. I didn’t enjoy that sex because I was like it is inside there, how long will this man take to come out and after that you go and check it is it still in that position or it will have gone too much in. So I had that fear on the first day. Then when I confirmed it was there, it was not tampered with, like it didn’t slide it just collected that blood till I was ready to go” (R8 G2).

“I inserted it, after inserting it he also just did the work then he asked me that you told me you are on your menses, where is blood? He didn't see it. I told him that is it true you have not felt it? You have not even felt something down here? ‘I didn’t feel’. I went and removed it and brought to him that “here it is, this is what I inserted. It is called menstrual cup, yah’? Then I said that this is a good thing let me go with it at work, so interesting. No one feels it at all. You know his brain is also not functioning, you are the one who will feel it because you are the one who had put it on but the client will never feel it” (R8 G4).

“I pretended I was unwell. ‘Lets just go but I am unwell’. He shouldn’t come with a lot of energy because I could be hurt, I told him I am sick, he told me ‘I want to take you slowly because you are sick’. Yes, I am sick he came from Nairobi. I will take you slowly, I said ‘okay’ but I was not comfortable. So I was moving a bit so that he doesn’t insert deeply (laughter) so that it doesn’t go straight, so that it goes on the sides, and he even told me today ‘you are truly sick, you are not as energetic’ When he was done, I went to the toilet and checked. I got I was ok” (R6 G2).

After these first experiences, the FSWs realised that they could wear a cup without a partner knowing about it.

“I would tell them [other sex workers] this thing is so comfortable, while having sex there is no feeling at all like the client cannot feel like there is anything inside. So I can encourage them they use that thing when having sex, its good, it’s the best” (R9 G2).

Only one FSW spoke of the cup being “displaced” but followed it up stating that she had not inserted it well; another reported that her boyfriend had felt it but then explained that he knew she was going to use it beforehand. A third regaled the group with the story that her client could feel it because they were having very rough sex:

“Yes, you know there are clients who like tossing here and there, so that day he felt it. He asked me, what are you wearing? I told him I am in my menses and he is the one enjoying this thing, he told me at the moment you women what do you do? So you have brought something that hides it, I can’t feel that you are in your menses, I told him yes. He felt it because the games were many [laughter] (R5).

“It was Gor and AFC Leopards [Football teams] [Laughter] (R2).

“He took it positively, so forced me we sit down I tell him. That this thing I am using so that I get my time with him even if I am in my periods but he took it positively” (R5 All FGD G2).

When asked about the cup in all client FGDs, only two men thought that they may have had sex while the cup was *in situ*, having felt what they described as “something.” When pressed further, one reportedly felt something “in her body” but did not feel able to ask her about it. Only during the FGD did he conclude that it may have been a menstrual cup. The other described his reaction as follows:

“Some I don’t feel but there is one that I have felt put it on. Mmm, now I don’t know whether her vagina was shallow or the penis was long {laughs} I asked her that ‘[name] I have felt something there, does it mean that, that is the deepness of your vagina or what is happening’? Then now she told me that I’d put it on. [And how did you feel about it? (Moderator)] You know it is a must … first I was irritated why so. And I thought she put it on the wrong way or the penis was long or what was happening. But after telling me, because I understand what menstrual cup is, I thought that this lady was attending [menstruating] just the way we said that some of them when they are attending that is when they feel like having sex. Now I thought, I just concluded that it is just okay, because that is the way she gets her daily income. Yah, I felt good because I realized that she was concerned with my life she didn’t want me to get dirty with the blood. Secondly, I also felt that it was not her wish but it’s life that made her to get into this because she also need money for survival when she is at work” (P4 FGD1).

All other clients reported that they had not had sex with an FSW who was wearing a cup, or more usually that they did not know if they had done or not.

### Secrecy

Few FSWs had told a boyfriend or client that they were wearing a disc, and those they told were carefully chosen beforehand anticipating that their reaction would not be negative or violent. One exception was R4, who enthusiastically stated:

“I have told three different men. And I told them it’s a very expensive thing, I told them I am not these cheap girls [Laughter], I am using something bought at 5000 kshs, they liked it, and I told them I do not use cheap things [Laughter]” (R4 G1).

Narratives then explored whether clients should be informed when FSWs were wearing a disc. Replies varied, with some unsure: “Should I tell him I have something in my vagina?” (R9 G1), but many responded negatively. A common perception was that clients, particularly new or not regular ones, should not be told, primarily because they might then leave without paying or use the information as an excuse to pay less.

“There is no need of telling a client that we have a cup. Because these people when they just feel, you just tell a client that you’ve put something inside there, eheee, that’s how he vanishes. Even if he was already erect, he wanted to … he freezes and that’s how the money leaves you, he will vanish. So there is no need of telling them” (R7 G4).

This point was echoed by several male clients when asked whether FSWs should tell them whether they were wearing a disc during sex. Those who were not in favour, over half of the men, generally felt that learning that a sex worker was on her menses would affect “morale,” a word used quite often, as was the phrase “it kills the mood”, and additionally, “I will just ‘deplete’.” As one succinctly stated:

“Because sometimes maybe I have the morale of wanting to have sex, and then you tell me something, you know, it kills the mood completely” (P8 FGD5).

A longer explanation was proffered in another FGD.

“you know like all of us, we have said that we are scared of blood, right. So by default when someone tells you, ‘Ah, I have a cup’, what will ring in your mind is the blood, that is what will ring in your mind. And you will find that the lady will even lose a client just by mentioning that to him, so like this is my what … whatever, my mind, when she has wearing it, let her just keep quiet about it”. He continued later asking; “Why do you want her to tell you? …… You see when you start interrogating what the chicken has eaten you will never eat that chicken if it is served to you” (P9 FGD6).

Those men who would prefer to be informed seemed unable to voice strong reasons for their choice, just making comments such as

“Let her just tell me, I don’t have issues let her just tell me”. [“You will not deplete”? (Moderator)] “No, let her just tell me, I will just continue with the act” (P6).

A similarly nebulous explanation was provided in FGD 8 where, unlike the other FGDs, the majority of men did want to be informed.

“You are going to wear a condom, right, when you are wearing a condom, she is aware that you have a condom on right? So she should tell me she is wearing a cup” (P4).

“Why does she not tell you that she is wearing that cup?” (P3).

“Any other reason why you would like to know?” (Moderator).

“I just want to know because I do not want to deal with something that I don’t know, it’s good to have the information so that I can feel free” (P4).

A few FSWs suggested that the cup may be perceived by clients as a barrier like the condom, as one woman explained further: “So that is why if you tell him, if you realize he is a client who feels that condom is acting like a barrier he does not feel the whole of you the way he wants. If you tell him again that you are wearing that thing, he will have to leave you and he will go away” (F3 G3).

Others thought that men simply would not understand what the menstrual disc was and hence it would be an unnecessary complication to involve them, particularly as they cannot feel it *in situ*. They could remain ignorant of the situation. A couple of FSWs argued that men's lack of understanding, along with misconceptions, could prompt a reaction of violence if they were told.

“You can tell a new one [client] and you can encounter blows on your face, that’s where violence begins. That’s when you will receive blows and he will be like … ‘why do you want to kill me’? Because they normally believe that we girls we are cunning. He says that ‘this girl is putting this thing on maybe she wants to infect me with HIV’” (R7 G4).

There was little indication from the men’s FGDs that violence would be a common male reaction to learning that an FSW was wearing a disc during their sexual activity. However, two males in FGD 9 reacted quite strongly, with one stating that if he found out “I will be pissed off” (P11), and the other saying, “I will quarrel her” (P2). When asked why, the second reported, “I am really scared of blood.”

It appeared that other clients could have misconceptions, prompting a negative reaction, although not directed towards the FSW herself, as FGD2 reasoned:

“So, I felt I would hurt her. I don’t think I would have sex with someone knowing she has got that stuff inside there,” and later “I will not do it” (P3), the phrase repeated by P7, P6, P5, and P1. When asked why:

“I feel I would hurt her” (P5).

“I think it’s' very tricky, unsafe. Very tricky. I can hurt her” (P1).

“I just feel that it can get stuck inside there” (P4).

Notably, P5 and P1 became more nonchalant on learning more about the menstrual disc and that it would not hurt the FSW.

“I am fifty fifty” (P5).

“I will just do it” [“Reason”? (Moderator)] “Reason being, at least that blood has been stopped, now I won’t be in contact with that blood. So, I don’t see any problem there” (P1).

P4, however, remained adamant about repeating his previously voiced concerns:

“You know sex is … because it’s being driven through my mind. And once you’ve seen something inside … You’ve just heard something is inside me … Mmm, morale disappears completely.”

Across all FGDs, however, there were some clients who reported that they would not be bothered whether a sex worker was using it as long as they did not know or could not feel it.

“If it does not harm me then I’ve no problem with it, let her just wear it. If its harmful …” (P3 FGD4).

“If sex will just be normal and she satisfies me, there is no problem” (P10 FGD7).

Although not mentioned by clients, some FSWs were more likely to believe that long-term partners such as boyfriends or regular clients could be told about the cup as they were often involved in paying or buying pads for the women and would therefore be happy to save money. As one FSW pointed out, not all were insensitive, saying, “Mmm, there are even some clients who are … a person who can just look at you and tell you like no, it seems today you are on your menses. Because you know some of us their faces usually change … if he sees you like today you just tell him that no, even if am on my menses, there is no problem because I am safe” (R9 G4).

### Sensitisation

Some FSWs altruistically suggested male clients could be informed so that they could then purchase a disc for their wives. Despite the majority not wanting individual clients to be told, many agreed that sensitising all men about the menstrual disc was a positive step forward. A few likened this to the female condom, where they reported that successful sensitisation had occurred over time and believed that the same could be achieved with the disc.

“You know, sensitization must be done, to everybody to be able to understand what are menstrual cups. And it takes time, and at the end of the day these are the men we meet on the ground you see. So they must be sensitized, same way sensitization ya female condoms was done. No it didn’t just start within a day that they have brought female condoms for people to use, they involved sensitization. At the moment also the same thing that was done, let be done for them to be able to understand, that I meet a woman in a club or I meet a client and I get there is something she is wearing, they will be able to understand that those are menstrual cups, and it is not something to harm them you see. They must know it is something that will not harm them and first and foremost both of us will enjoy because I will have gotten the money and he will have gotten the services that he wanted” (R5 G2).

“Mmm, we can use these men, you know when men are also trained, if they are also given the right information, they can also go and spread it. Because when they are also told the benefit of menstrual cup, it will also force them that when they go to the ground, they talk about its benefit too” (R9 G4).

With only one exception, wherein an FSW who used the disc but stated that she was still a little afraid of using it, our discussions concluded with many positive remarks about it. The following excerpt from G2's discussion amongst FSW participants illustrates responses when the moderator asked their future intention on using the cup.

“Once the 10 years elapse, I will try hard and get another one because I love it. I think it is only menopause that will make me stop using the cup” (R6). [laughter] “Me too, once the 10 years are over, I will have to get another cup until I stop having periods” (R9). “Until I stop bleeding” (R5). “ I cannot stop using the cup until I reach menopause and even if it gets lost, I will look for it and buy it, I love it so much” (R4). “After the 10 years, we need to have another one because one cannot use it anymore. It may get lost, it can fall in the toilet. You will be forced to get another one” (R7).

## Discussion

Recognition and use of the menstrual cup is growing in low- and middle-income countries. However, the literature is scarce around women's experiences of using a disc-shaped menstrual cup, especially during sexual intercourse. For this reason, our discussion is able to make a comparison with studies that only use a bell-shaped menstrual cup. Our findings indicate that the menstrual disc is a very acceptable and preferred method of menstrual hygiene management for FSWs, with the additional benefit of enabling sex during menses without the FSWs harbouring any concerns about menstrual blood.

Both initial reactions and early experiences of FSWs using the menstrual disc in our study mirror studies of girls and women using menstrual cups despite differences in the overall shape and size (18, 19). Initially, there was some surprise and apprehension, including about its size and how it would fit, with concerns about inserting it for the first time and whether it would stay in place. As with previous studies, some FSWs needed repeat attempts to insert it correctly, with some reporting initial pain and/or discomfort, but all of them recounted that they continued to try using relaxation or better positioning techniques until they were successful. Our results suggest that the FSWs were more persistent in their effort than the girls and women reported in the majority of studies, a finding corroborated in this main trials survey data (not shown), where 94% of FSWs reported using the disc successfully by the end of the first month. In contrast, other studies (2, 5, 7, 20) reveal that not all girls and women given a cup will ever try using it or make repeat attempts until they become successful, ultimately resulting in lower and slower overall uptake.

That all FSWs were determined and successful in using the menstrual disc may be attributed to the study methods employed (i.e., the training curriculum, the monthly check-ins, and the peer support model) or the population type. Studies have suggested that peer support can be vital in the early stages of menstrual cup adoption and mastery to optimise uptake and use (2, 5, 7, 20). We provided a single, 2 h group training session to the FSWs, and then for the first three months after they received the disc, a study staff member called each participant to assess disc use and identify any associated problems, talk through any issues, and answer any queries, whilst FSW peers and study participant colleagues were also on hand to troubleshoot any insertion and removal issues. For participants who reported any difficulties in insertion and removal, they were provided the option of refresher training over the phone or in person with the coordinator. It should also be noted that many of the participants knew each other from the community or through the community-based organisations they were registered with, and this encouragement and reassurance contributed to a particularly strong and supportive network. Finally, our participants may have differing needs to the general population, which particularly draws them towards adopting the menstrual disc for menstrual hygiene. For instance, menstrual discs enable them to continue working and thus earning during their menses, an important consideration amongst women who rely on sex work for their livelihood.

Once the technique of wearing the disc was mastered by the FSWs, most of the challenges they faced were similar to those reported in other studies for example, dropping the disc (21, 22) and the lack of privacy to empty and change when washrooms were not available (18). Although the latter could apply to changing any method of menstrual hygiene, it seems to be noted as more of an issue when wearing a cup or disc due to the length of time and privacy needed to do this. To our knowledge, other studies did not mention about the extra care that women need to take (and seemingly often did) when drinking or taking drugs. This may be due to the fact that the majority of studies were conducted in populations who are less likely to drink heavily or take drugs (e.g., adolescent schoolgirls) (19), or less likely to feel comfortable reporting these behaviours, or the subject not being raised by a moderator or asked in a survey due to cultural insensitivity or inappropriateness (23, 24). Drug and alcohol use are common amongst FSWs (25, 26), and our participants felt comfortable during the group discussions to talk about these issues. The FSWs in our study provided successful solutions, for example abstaining from or reducing alcohol and drug use during menses, suggesting that many took seriously concerns about wearing the disc too long, valued their disc, and wanted to avoid dropping or losing it.

Once insertion was mastered our participants praised the menstrual disc. As with most cup studies (2, 18, 19, 23), users compared it favourably to other methods (usually disposable pads) in relation to financial benefits, comfort (i.e., less itching, chafing and bruising), and reduced leakage. For comparative reasons it would appear that our FSW prefer to use the disc than their previous method of menstrual hygiene, usually pads. We were surprised that freedom from using harmful practices to hide menstruation or any associated improvements in hygiene were mentioned by just a few women suggesting they were not a particularly important consideration or perceived benefit of using the disc amongst our participants. It may be that these benefits were simply outshone by the other advantages noted, whilst we, as researchers are more attuned to concerns around unsafe practices.

The physical safety of FSWs engaging in sex with clients when menstruating is a matter of concern. Our previous studies (10) (see text footnote 1) highlighted issues around violence or non-payment should a client discover that the FSW was menstruating. In these studies, we found that some clients admitted to becoming violent or reported to have become violent to the FSW or even refused to pay money to them. In the current study, it was important, therefore, to ascertain whether the FSWs were not put at additional risk if a client became aware that they were menstruating and wearing a menstrual disc. However, apprehensions that the partner would “feel” it were *mostly* unfounded. We have italicised “mostly” as the few instances reported by an FSW could be explained by the partner knowing beforehand (which may have coloured his judgement) or poor placement so that it became dislodged during “rough” sex. Similarly, the two instances of a client feeling “something” during sex were not conclusive of it being the disc. We were unable to assess objectively for ethical reasons whether any clients would have had sex with an FSW who was wearing a disc. However, because most of the FSWs were using it with their clients, and many of the male participants were referred to the study by the FSWs, lead us to conclude that some clients had most likely been exposed but unaware that a disc was being used. Importantly, in the few instances reported of possible awareness, there were no adverse reactions by clients.

Although initially not wanting to tell individual clients unless they were regular ones, the FSWs were in favour of men in general being informed about the menstrual disc. This finding aligns with clients themselves who were generally accepting of the disc in theory and wanted to know more about it. Our prior study (10) also found that when clients were informed about the menstrual disc, they responded positively, accepting it as a safe, hygienic, and cost-effective alternative. However, wanting knowledge about it appears to be only a general and theoretical curiosity, conditional on remaining ignorant of it being worn at the time of intercourse. This response reflects negative client views on menstruation, which some reported would affect their libido, also expressed by male clients in our prior work (10). As a result, many FSWs and clients alike suggested sensitisation programmes for men, which would also help to remove the misconceptions that clients had about the disc and mitigate some of their negative reactions.

We note possible limitations to our findings. Because women were given menstrual discs as part of the study, it is possible that the participants felt some obligation to vocalise a positive opinion. However, the enthusiasm with which the participants spoke, both during the FGDs and as part of peer support networking, suggests that this is a genuine feeling. Similar content and expressions were evident within and across the four FGDs (hence we felt saturation was achieved earlier than anticipated), which reassures us that the data are reflective of participant opinion. Furthermore, the data were also substantiated by the quantitative surveys conducted in parallel, which will be published at the time of completion of the study.

## Conclusion

In this study, the menstrual disc was reported as an acceptable and preferred method of menstrual hygiene for FSWs, reducing the discomfort and leakage associated with other products. Women reported financial benefits in relation to monthly outlay for absorbents and continued with their work during menses. Views of the FSWs suggest that the disc can be worn comfortably during sex and without leaking. Importantly, the FSWs reported that clients for the most part appeared to be unaware that they were wearing it during sex, and whilst we could not confirm with certainty that the clients were indeed unaware, their narratives appeared to substantiate the FSW reports. The findings suggest that the use of the disc would not engender more violence. Our findings also indicate that early support and encouragement is needed for FSWs, and this could be instrumental in determining the success of menstrual disc take-up. Taking into account the issue of sensitisation of men as part of scale-up could generate further support and help to create awareness about menstrual health and hygiene.

## Data Availability

The datasets presented in this study can be found in online repositories. The names of the repository/repositories and accession number(s) can be found below: https://doi.org/10.25417/uic.31040881.

## Footnotes

^1^McCullough E, Zulaika G, Osire E, Akinyi C, Otieno FO, Phillips-Howard PA, et al. A qualitative exploration of perceptions and potential impacts of menstrual cups among female sex workers in a peri-urban area of western Kenya. Dept of Clinical Sciences, Liverpool School of Tropical Medicine / Rush Medical College (2026).
