# Supplementary material for: “Should I tell him I have something in my vagina?” Female sex workers’ perceptions and experiences of using a menstrual cup, and client reactions: A qualitative study in Western Kenya
Source: Front Reprod Health. 2026 Feb 2;7:1740096. doi: 10.3389/frph.2025.1740096 (PMC12907317; doi:10.3389/frph.2025.1740096)
Supplement: Supplementary file 1 [file Table1.docx]

**Women –phase 2 after intervention**

- **Warm up – initial thoughts on menstrual cup -**What did you think about the menstrual cup when you first learned about it / we first showed it to you? Who has tried the cup? Who hasn’t? How did you find using the cup? Is there anything about the cups that you don’t like? Is there anything you do like about the cups?
- **Views on training / information -**What did you think about the training / information you were given about the cup? Did you need more information – on what? Would it have been better if it was given to you differently – in what way? (person, context, delivery mode, understanding) Were all of your questions answered at the time? What did you want to know more about? Was there anything we spent time on that was not needed or not done in a way that was helpful? What?
- **As the intervention went on, more women were coming to us already informed on the cup by their friends, coworkers, and peers.** Were you informed on the cup before you received the training with us (NRHS/Edyth/Cynthia), and if so who did you hear about it from? Was the information shared by your friends/co-workers/peers similar to or different from the training we provided? In what ways was it helpful to hear from your community before having the formal training? Do you think it would work to have peers do all of the training? Why or why not?
- **Experiences of the different aspects of cup use -**How did you feel just before the very first time you used the cup? Why do you think you felt this way? What is your experience of inserting the cup the very first time? What could have helped at the time? And now? Any problems / what / why? Is there anything you need or do to make it easier to insert or empty? What is your experience of cleaning it? Is it easy or problematic? Why? Is there anything that would make it easier to clean? Have you dropped it at all – where / why? Do you think this may be a problem in using the cup?
- **Non – use of cup** Have you or anyone you know anyone who stopped using it? Or who did not try to use it? Why? Do you or anyone you know of, use it sometime but not always? Why? When? Do you think there is anything that can be done to help women start using it/reusing it?
- **Others and cup use** Has anyone asked to use your cup? Why? What happened? Did you or anyone you know of give the cup away or sell it? Why? What happened? Did you or anyone you know have your cup stolen or taken without your permission? What happened? (probe: did the person use it, did you get the cup back)
- **Comparison of cup with previous method -** How do you feel about using the cup when you’re not engaged in sex work? Is it easier to keep yourself clean now with the cup, or with what you were using before? Do you think your vaginal health has changed? If so, in what way/s? (probe, discharge, odor, itching)
- **Use and experience of cup during sex [note to moderator: ensure this question is covered slowly, with ample probing and discussion]-** Have any of you been using the cup during sex with a client/s? Why / why not? What was your experience of this? (prompt – ease, comfort, leakage, removal, clean). Is there anything that you do that makes it easier or better to wear during sex? What advice would you give other women about wearing a menstrual cup during sex?
- **Client view of cup use -** Did your client/s know you were wearing? Why? Why not? (did you discuss before or after, could they tell you were wearing it – why) If yes: what was their reaction/s? If no, what do you think their reaction might have been, is there anything that could be done that might make it easier to tell them? Which partners do you tell? Why these? Are they any types of partners who you would not tell? Do you think they should know? Why / why not? If menstrual cups were to be scaled up to the sex worker community, what work – if any - should be done with male clients?
- **Impact on livelihood -** If you have been using it during sex with clients, has this made any impact on your work practices? In what ways? Do you take more clients than you used to? If you have increased income now with cup use, how do you use that money?
- **Drinking or drug use:** Have you or any women you know experienced challenges to cup use when they are drinking or using drugs? If yes, what types of challenges did women experience when using the cup while drinking or using drugs? Did drinking or drug use interfere with remembering to remove and empty the cup? Cleaning it properly? Dropping or losing it?
- **Places of sex work:** Are there certain sex work hotspots that are easier or more difficult to use the menstrual cup? For women who work in brothels or bars or guest houses, what role do places of work have in supporting menstrual hygiene? What could be done to facilitate menstrual management for women who work in these settings? For women who work on the street, what are challenges with menstrual cup use? What could be done to facilitate menstrual management for women who work on the street? For places where access to water is a barrier: do you carry water with you? Why or why not?
- **Abuse and Violence:** Now I would like to change topic a bit, thought it is still related. How are abuse and violence currently affecting the sex worker community? By abuse, we mean physical, emotional, or financial – all types of abuse. What types of abuse do women in the sex worker community face? Do you think the abuse has changed over the past few months, that is since the beginning of 2025? In what ways? (probe: are types of abuse changing? Frequency of abuse changing? Are the perpetrators of abuse changing? If yes: in what way/how are they different?) Why do you think abuse (frequency, types, perpetrators) is changing or not changing? Do you think that having or using a menstrual disc or being part of this study has affected levels of abuse experienced? (If yes: why do you think this?)What do you think can be done to prevent abuse towards sex workers?

**If participants do NOT identify changes in abuse**: In the last survey we found that a lot more women reported to us they had been abused than previous surveys - why do you think this has changed?
