# Supplementary material for: “Should I tell him I have something in my vagina?” Female sex workers’ perceptions and experiences of using a menstrual cup, and client reactions: A qualitative study in Western Kenya
Source: Front Reprod Health. 2026 Feb 2;7:1740096. doi: 10.3389/frph.2025.1740096 (PMC12907317; doi:10.3389/frph.2025.1740096)
Supplement: Supplementary file 2 [file Table2.docx]

**POWWER Health Series 2 Focus Group Discussions**

**Men’s Guide**

**Economy & Financial Status**

- How do men like yourselves earn a living? What types of jobs are they working?
- In the past several months, is it easier or harder to earn enough money to live off? Why? Probe: Is this to do with fewer jobs around / being paid less / costs of things have gone up?
- Are there things you are having to go without that you would like or used to have / buy? (If yes; What sort of things? How does this make you feel?)
- What about having money for family / wives / girlfriends – do you currently spend less on them than you used to? (If yes: Why? What would you like to buy them or pay for that you can’t?)
- What about having money for sex workers? Are you currently spending less on them than you normally would? (If yes: Why? In what ways are you spending less – such as, Are you having fewer visits to sex workers because you have less money? Are you paying them less? Are you having to go to different venues or have sex with women who charge less’

**Stress and Mental Health**

(Depending on situation described above regarding work / lack of money, link what they have talked about)

- Does this (reductions in employment/finances) cause you stress? (If yes: in what way/s does stress have an impact on you? How does this make you feel?)
- Do any other sort of things cause stress for you? (If yes: What things? Why? How does this make you feel?)
- Do you think stress causes you to behave differently? (If yes: In what way? Does this behaviour affect your family/wives/girlfriends? How?)
- Does stress affect the way you behave with sex workers? If yes: in what ways?
- What do you do to make yourself feel better or forget about your stress? (Does it help? How? Why?)
- Aside from what you currently do to help yourself feel better, **What else** would help with your stress and mental health? Meaning, what do you **wish** for, to help you manage your stress? (What are barriers to/ Why don’t you / can’t you do / use this?)

**Abuse**

- Who has heard of the term ‘gender based violence’…what do you think this is? Who has not heard of gender based violence?

Probe: for only men to women? (If they are only discussing GBV as only physical violence) Are there other types of abuse that are gender based violence? What kinds? RAPE (Why do some men rape)

- What forms of GBV do you think are most common around here? (Why these / this?)
- What are the situations that might make people more likely to commit GBV in this community? (Probe for example, poverty, culture, social status, etc.?)
- Has the problem of GBV in this community become worse, better, or stayed the same in the last 6 months? Which types of GBV have become worse, better, or stayed the same. Why do you think this / these have become worse/better? (probe: economy, mental health, awareness/society’s views of violence, laws)

**Interactions with women and sex workers**

- What do you think about a man being violent to a woman? Do you think this is (ever) ok? (Probe: husband/wife: what the act is: Push / slap….fist / weapon…sexual / rape? ) When (what type of instances) might it be ok? What about if the woman is menstruating? When (what type of instances) might it not be ok? Probe what the act is: Push / slap….fist / weapon…sexual / rape?)
- What about with sex workers? Do you think this is (ever) ok? (Probe: what the act is: Push / slap….fist / weapon…sexual / rape?)When (what type of instances) might it be ok? When (what type of instances) might it not be ok? Probe what the act is: Push / slap….fist / weapon…sexual / rape?)
- What do you think about these men? What might make them violent? Is it their fault that they are violent? Can you give reasons for your answer? Do you think that it might be the fault of the woman? Why? why not? Does it matter if it is wife/girlfriend or sex worker? What about if the woman is menstruating?
- What do you think about a woman being violent to a man? Do you think this is (ever)ok? (When/what circumstances might it be ok? Probe: husband/wife; what the act is: Push / slap….fist / weapon…sexual / rape?) When/what circumstances might it not be ok? Probe on type of act Push / slap….fist / weapon…sexual / rape?)
- What do you think about these women? What might make them violent? (probe: economy, stress/mental health, alcohol, drugs) Is it their fault that they are violent? Can you give reasons for your answer? Do you think that it might be the fault of the man who is being hurt? (Why? /why not?)

**Interactions with sex workers**

- Thinking over time, since last year, have men’s interactions with sex workers changed or stayed the same? For example, changes in how much they are paying, the types of sex acts they are doing, where they are meeting sex workers? (Probe: Changes in condom use, anal sex, rough sex, sex during menstruation) Why do you think this is?If any changes: What do you think is causing these changes? (Probe: economy, mental health, women’s behaviors)
- Do you think that the way men treat sex workers has changed in the past 6 months? Has it gotten better or worse? In what ways? (If change: Why do you think it’s changed?)
- What about the how sex workers are treating the male clients? Do you think it has changed in the past several months? Has it gotten better or worse? In what ways? (probe: cost of sex act, use of condoms, types of sex act, availability of women, sex during menstruation, less access to services or care) Why do you think this has changed?
- Have you ever heard about sex workers stealing from their clients? Under what circumstances does this happen? Why do you think it happens? Has it changed in the past 6 months – more frequently, less frequently, no change? If yes: why do you think it has changed?
- How do clients react if they find that the sex worker has stolen from them? (probe: yelling, beating, revenging, involving police) How do the women react if they are caught stealing?

**Menstrual health**

As you may know, we are doing a study to support sex workers during menses, by providing them menstrual cups that can be worn during sex. Approximately one year ago, we have given menstrual cups to over 300 women.

- To your knowledge, have you had sex with a sex worker while she was wearing a menstrual cup? If Yes, how did you know she was wearing a menstrual cup? (Probe: She told you, you felt it, you saw it) If Yes, how did you feel about the woman wearing a menstrual cup during sex? (Probe: Did you feel safer from disease, cleaner, angry, deceived?) .If Yes, what type of relationship with the sex worker – you were her regular client or a first time client?/How would you feel if you found out a woman was wearing a cup /would you like to know or be told she is wearing a cup if no how would you feel about knowing you slept with a woman wearing a cup/would you like her to tell you that she is wearing the cup and why

**Closing remarks**

- Is there anything we have not talked about today that you think is important to mention - related to the economy, men’s stress and mental health and impact on their interactions with women?
- Is there anything else you would like for us to know about that could help men like yourself?
